# Low-Risk Antenatal Care Enhanced by Telemedicine: A Practical Guideline Model

**DOI:** 10.1055/s-0042-1753505

**Published:** 2022-07-19

**Authors:** Talita Colombo, Lorenza Bridi Todeschini, Mariana Orlandini, Hallana do Nascimento, Franciele Cordeiro Gabriel, Rafael José Vargas Alves, Airton Tetelbom Stein

**Affiliations:** 1 Postgraduate Program in Health Sciences, Universidade Federal de Ciências da Saúde de Porto Alegre, Porto Alegre, RS, Brasil

**Keywords:** prenatal care, telemedicine, teleconsultation, management guideline, systematic review, pré-natal, teleconsulta, telemedicina, diretriz clínica, revisão sistemática

## Abstract

**Objective** To develop a protocol for hybrid low-risk prenatal care adapted to Brazilian guidelines, merging reduced face-to-face consultations and remote monitoring.

**Methods** The PubMed, Embase, and Cochrane Library databases were systematically searched on telemedicine and antenatal care perspectives and adaptation of the low-risk prenatal care protocols recommended by the Ministry of Health and by the Brazilian Federation of Gynecology and Obstetrics Associations.

**Results** Five relevant articles and three manuals were included in the review, for presented criteria to develop this clinical guideline. We identified, in these studies, that the schedule of consultations is unevenly distributed among the gestational trimesters, and ranges from 7 to 14 appointments. In general, the authors propose one to two appointments in the first trimester, two to three appointments in the second trimester, and two to six appointments in the third trimester. Only three studies included puerperal evaluations. The routine exams recommended show minimal variations among authors. To date, there are no validated Brazilian protocols for prenatal care by telemedicine. The included studies showed that pregnant women were satisfied with this form of care, and the outcomes of interest, except for hypertensive diseases, were similar between the groups exposed to traditional and hybrid prenatal care.

**Conclusion** The presented guideline comprises the Ministry of Health recommendations for low-risk prenatal care and reduces exposure to the hospital environment and care costs. A randomized clinical trial, to be developed by this group, will provide real-world data on safety, effectiveness, satisfaction, and costs.

## Resumo

**Objetivo** Desenvolver uma diretriz clínica híbrida para atendimento pré-natal de baixo risco, mesclando consultas presenciais e remotas por telemedicina, adapta às recomendações brasileiras.

**Métodos** Revisão sistemática da literatura nas bases de dados PubMed, Embase e Cochrane e adaptação dos protocolos de atenção ao pré-natal de baixo risco preconizados pelo Ministério da Saúde e pela Federação Brasileira das Associações de Ginecologia e Obstetrícia.

**Resultados** Cinco artigos relevantes e três manuais foram incluídos na revisão por preencherem critérios para o desenvolvimento desta diretriz clínica. Nos estudos incluídos, identificou-se que o cronograma de consultas se distribui de forma desigual entre os trimestres gestacionais, variando entre 07 e 14 encontros. De forma geral, os autores propõem uma a duas consultas no primeiro trimestre, duas a três consultas no segundo trimestre e duas a seis consultas no terceiro trimestre. Somente três estudos incluíram avaliações puerperais. A rotina de exames preconizada apresenta mínimas variações entre os autores. Até o momento, não existem protocolos brasileiros validados para atendimento pré-natal por telemedicina. Os estudos incluídos evidenciaram a satisfação das gestantes em relação a esta forma de atendimento, e os desfechos de interesse, excetuando doenças hipertensivas, foi semelhante entre os grupos expostos ao pré-natal tradicional e ao pré-natal híbrido.

**Conclusão** A diretriz apresentada contempla as recomendações do Ministério da Saúde para atendimento pré-natal de gestantes de baixo risco, reduz a exposição ao ambiente hospitalar e os custos de atendimento. Seu emprego em um ensaio clínico randomizado, a ser desenvolvido por este grupo, proporcionará dados de mundo real, relativos à segurança, efetividade, satisfação e custos.

## Introduction

Pregnancy is a period with important physical, psychological, and social changes. Prenatal care, defined by the World Health Organization (WHO) in 2015 as recommended medical and nursing care during pregnancy, aims to follow changes and identify early deviations from normality, thus allowing appropriate care. 1 2 Prenatal care should begin as early as possible, aiming to screen potentially harmful medication and behavior such as drug and alcohol use, smoking, and occupational chemical exposure; to develop educational and preventive actions; and to promote easy access to quality care. 3

In Brazil, low-risk prenatal care follows the recommendations of the Ministry of Health, which prescribes at least six prenatal consultations for pregnant women at low-risk. This is usually provided at the primary health care unit, although there is a tendency for it to be conducted at in-home visits. Whenever possible, the consultations should be performed according to the following schedule: up to the 28th week - monthly; from the 28th to the 36th week - biweekly; from the 36th to the 41st week - weekly. The higher frequency of visits at the end of gestation aims to assess the perinatal risk and clinical-obstetric complications that are more common in this trimester. There is no discharge from prenatal care before delivery. 4 5

These recommendations were implemented by the Prenatal and Birth Humanization Program in 2000, and this guidance is still being performed. 6 However, the ideal number of appointments in low-risk prenatal care remains controversial. The pandemic by coronavirus disease 2019 (COVID-19) and the need for social distancing forced health providers and health systems to redesign access to care. 1 Telehealth emerged as a strategy to solve many of these challenges imposed by the pandemic at the local level, including prenatal care. 2 During the pandemic, The Royal College of Obstetricians and Gynecologists (RCOG) recommended the use of telehealth whenever possible to minimize the frequency of visits to the healthcare service. 3 Similarly, the American College of Obstetricians and Gynecologists (ACOG) and the Society of Maternal-Fetal Medicine (SMFM) issued guidelines on prenatal care during the COVID-19 pandemic, including testing and modification of traditional prenatal consultation guidelines, with the use of telehealth in areas in which the COVID-19 epidemic curve was not, and there was a need to reduce the access to face-to-face medical visits. 4 The COVID-19 pandemic increased the urgency of determining the ideal timing and frequency of prenatal care. It also further highlighted the need to define criteria on which visits are acceptable to be performed via telemedicine, with a particular focus on maternal and child outcomes and maternal preference. 5

Brazil was reluctant to regulate the practice of telemedicine in a broad and definitive way, causing many doubts and insecurity about its practice. Nevertheless, the crisis caused by coronavirus rushed the publication of the Law n. 13.989, of April 15, 2020, which determines the implementation of telemedicine during the crisis caused by the coronavirus, as there is a regulation that has authorized its use, in a very broad manner, since it is open to all forms of assistance, research, prevention of diseases and injuries, and health promotion. In Annex 1 , we detail the terms most commonly used terms in telemedicine. 6 7 8

Nevertheless, determining the ideal timing and frequency of prenatal care consultation is an important issue. Is also further highlighted the need to understand which consultations are relevant to be performed via telemedicine, and there is a need to focus on maternal and child outcomes as well as on maternal preference. 5 Therefore, this study aims to develop a guideline for hybrid low-risk prenatal care, adapted to Brazilian guidelines, merging reduced face-to-face consultations and remote monitoring,.

## Guideline Scope and Target

The COVID-19 pandemic has shown the requirement to adapt the Brazilian prenatal care model to ensure coverage, even in challenging socioeconomic situations, taking into account social characteristics that are relevant for the prognosis of the pregnancy outcome. The development of a guideline for hybrid prenatal care, in which face-to-face and remote consultations are available, that can provide early access, as well a regular schedule of prenatal care visits in a safe way, as recommended by the Ministry of Health. This model can result in costs reduction and lower risks; and it is expected that the same effectiveness and quality will be offered.

## Audience

The target audience for the recommendations in our guideline includes family-physicians, obstetricians, midwives, and policy makers who inform patient decision-making, clinical practice, and health-policy decisions.

## Disclaimer

This guideline is not applicable for all potential clinical circumstances. This guideline is not intended to supplant clinician judgment, and its recommendations should not be mandatory. For all recommendations, we have considered the certainty of evidence, patients' values and preferences, resources required, equity, acceptability, and feasibility. Clinicians are encouraged to apply the key strong recommendations, according to the clinical context of each individual patient, in which patients' values and preferences are taken into account.

## Development of Recommendations

The development of this guideline followed the recommendations of the Ministry of Health and the Brazilian Federation of Gynecology and Obstetrics Associations (FEBRASGO) for prenatal care. 6 The appropriateness of telemedicine care was based on the findings of the systematic review on the use of telemedicine in prenatal care, which has been developed in the last 5 years. To assess the certainty of the evidence available in the literature, the Grading of Recommendations, Assessment, Development and Evaluation (GRADE) system was applied, which classifies the quality of the evidence or its degree of certainty into four categories (very low, low, moderate, and high). 9 A detailed description of these steps can be found in the methods section.

## Methods

### Questions and Outcomes of Interest

Three sets of questions had been defined: regarding prenatal visits distributed as face-to-face consultations and teleconsultations and maternal and perinatal outcomes.

**Question 1** : How should the distribution between face-to-face consultations and teleconsultations be in a hybrid prenatal care?
**Question 2** : Which maternal outcomes should be assessed to ensure the effectiveness of the intervention?
**Question 3** : Which perinatal outcomes should be assessed to ensure the effectiveness of the intervention?

To identify studies addressing the questions of interest, a systematic review was performed. To encompass all topics, the participant, intervention, control, and outcome (PICO) question for the review was structured as: participants— *low-risk pregnancy and postpartum women* ; intervention— *hybrid prenatal care* ; control— *in-personal prenatal care* , and outcome— *obstetric and neonatal outcomes* . Settings are not applied to do a broader strategy.

### Literature Search

An overview of clinical trials that analyzed the association between teleconsultation and antenatal care was performed following the guidelines outlined by the Cochrane Handbook. 7 It is reported according to the Preferred Reporting Items for Systematic Reviews and Meta-Analyses (PRISMA) guidelines. 8 The electronic search was performed in the following databases: PubMed, Embase, and Cochrane Library. The searches were performed on July 26, 2021. Medical subject heading (MesH) terms and entry terms were related to low-risk pregnancy, antenatal care, prenatal telemedicine, traditional prenatal care, and obstetric outcomes. We adopted a high-sensitivity strategy, with no restrictions on study design, language, and publication date. The terms combination adopted for search strategy performed in PubMed included MesH terms regarding low-risk pregnancy; low risk prenatal care; traditional prenatal; telemedicine prenatal care; and obstetric outcomes. The search strategies applied in the other database are available as Supplementary Material ( Chart S1 ).

### Eligibility Criteria

Any peer-reviewed article published and addressing a research question relating telemedicine and in low-risk pregnancy and postpartum women was eligible for inclusion. Editorials, commentaries, posters, and preprint articles without peer-review until the last review were excluded.

### Study Selection

All search results were imported into Rayyan, a web app for systematic reviews. 10 Two reviewers (T. C. and L. T.) screened the titles and abstracts of literature independently, and any disagreements were solved by consensus or by a third reviewer (M. O.). Two independent reviewers read the full text of the selected articles to confirm their eligibility (T. C. and L. T.). In the case they fulfilled the inclusion criteria in the overview, these data were extracted from each meta-analysis independently. A standard form was created in Google Forms (Google LLC, Mountain View, CA, USA) and was extracted by two reviewers (M. O. and H. N.). Any disagreements were resolved by consensus or by a third reviewer (A. S.). In these steps, all authors independently received all selected studies and performed the full text reading, confirmed their eligibility, and extracted the data.

### Data Extraction

Information about the publication (author, year, country), details of the methods of clinical trial (inclusion and exclusion criteria, intervention schedule, outcomes), and results (number and general features of participants as well as number of in-person and teleconsultations) were extracted independently by two reviewers using a standard form developed in Google Forms. Data about risk of bias and quality of evidence were also extracted independently by two reviewers using the ROB-2 and ROBINS-I tools. 9

### Risk of Bias

The risk of bias was evaluated with the RoB-2 and ROBINS-1 assessment tools in only three articles that were deemed appropriate for these analyses. In the study by Pflugeisen and Mou (2017), 11 a cross-sectional analysis was performed; in the study by Pflugeisen et al. (2016), 12 the patients could choose traditional or hybrid prenatal care; therefore, evaluation of risk of bias by Rob 2 or Robins I was not adequate. The limitations of the studies are presented on Tables 1 and 2 .

**Table 1:** Risk of bias 2 assessment tool

	Randomization process	Deviations from the intended interventions	Missing outcomes	Mesurement of the outcomes	Selection of the reported results	Overall bias
Tobah (2019) 10	Low	Some concerns	Low	Low	Low	Some concerns

**Table 2:** Robins I assessment tool

	Bias do to counfounding	Bias in selection of participants into the study	Bias in measurement of intervention	Bias due to departure from intended intervention	Bias due to missing data	Bias in measurement of outcomes	Bias in selection of reported results	Overall bias
Meza-Santibañez et al. 13	Low	Not informed	Not informed	Not informed	Not informed	Serious	Not informed	Not informed
Palmer et al. 14	Low	Low	Low	Low	Some concerns	Low	Low	Low

## Results

### Systematic Review

**Fig. 1:**
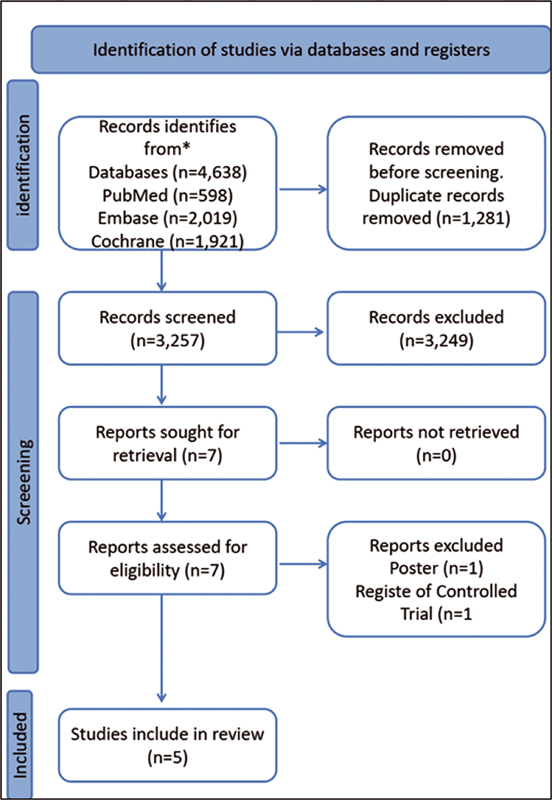
PRISMA flow diagram of included studies.

In our initial search, we identified 4,538 articles and documents. After removing duplicates and screening by titles and abstracts, 158 articles were fully read and subjected to the eligibility criteria; we identified five randomized control trials (RCTs) comparing hybrid prenatal care and traditional prenatal care in low-risk pregnant women. The PRISMA flow diagram maps out the number of records identified, included, and excluded, and the reasons for the exclusions ( Fig. 1 ).

Three studies were developed in the USA, one in Australia, and one in Peru, between 2011 and 2021, in-hospital antenatal care. The main characteristics of the studies are described on Table 3 .

**Table 3:** Summary of included publications

Periodic/Year	First author	Country	Participants	Study period	Design and details	Population characteristics	Outcomes of interest
American Journal of Obstetrics and Gynecology/2019	Tobah 10	USA	300 (T *n* = 150 H *n* = 150)	Sep 2011–Dec 2011	Randomized clinical Trial. T = 12 onsite appointments. H = 8 onsite appointments and 6 virtual visits; supplemented with wereables.	Low risk pregnant patients Aged 18–36 years. Obstetric tertiary center	Evaluate the acceptability and effectiveness of the hybrid prenatal program (OB Nest). Maternal and fetal outcomes of interest also were evaluated.
Am J Matern Child Nurs/2016	Pflugeisen 12	USA	1058 (Tn = 941 Hn= 117)	May 2011–Dec 2013	Clinical trial, without randomization Patients could choose between T and H prenatal care. T = 14 onsite appointments H = 9 onsite appointments and 5 virtual visits.	Low risk pregnant patients. Mean age T = 29.1 Mean age H = 30.3. Obstetric tertiary center	Evaluate demographic variables, preg- nancy and birth outcomes, and use of the health system.
Lancet/2021	Palmer 14	Australia	23.008 (T *n* = 20.031 H *n* = 2977)	Jan 2018–Mar 2020	Non- randomized study. An uninterrupted time series.	High and low risk pregnant Age T = 31–29 (5.19) Mean age H = 31–61 (5.04). Publicly maternity, two secondary and one tertiary referral hospitals	Comparison between traditional prenatal period and hybrid prenatal care in several factors (preeclampsia, stillbirth, CIUR…).
Rev Peru Ginecol Obstet/2021	Meza-Santibañez 13	Peru	NA	May 2020–Dec 2020	Descriptive study, theorical model.	High and low risk pregnant No patients enrolled.	Describe the new hybrid prenatal program with telemedicine of the Instituto Nacional Materno-perinatal
Matern Child Health/2017	Pflugeisen 11	USA	1,173 (T *n* = 795 H *n* = 378)	Mar 2013–Jan 2016	Cross-sectional study	Low risk pregnant Age T = 31.2 ± 4 Mean age H = 31.5 ± 5 Obstetric tertiary center	Check satisfaction in patients who received a hybrid model of prenatal with teleconsultations and those who received traditional prenatal care

Abbreviations: H, hybrid prenatal care; T, traditional prenatal care.

### Schedule of Visits

**Fig. 2:**
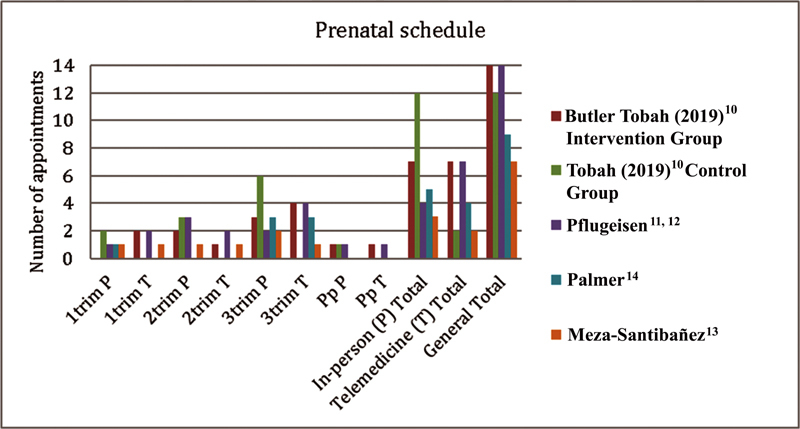
Schedule of appointments. P: in-person; T: telemedicine; Trim: gestational trimester.

Regarding the distribution between in-person and telehealth appointments, all studies proposed unequal proportions among gestational trimesters, ranging between 7 and 14 meetings. In general, the authors propose one or two meetings in the first trimester, two or three meetings in the second trimester; and two to six meetings in the third trimester. Only three studies included postpartum visits (one or two) ( Fig. 2 ).

## Discussion

### Maternal and Fetal Outcomes

The maternal outcomes evaluated included cesarean rates, pregnancy complications - as preterm birth and hypertensive diseases of the pregnancy. The reduction in number of in-person and emergency visits and the satisfaction of patients and health care providers with the assistance provided were also assessed in some of the articles.

In those studies that evaluated cesarean rates, there was no difference in the statistical significance between the groups that received the traditional antenatal care and the groups that received the intervention with telemedicine. In the North American study of 2019 by Tobah et al., 10 the control group had 14.9% of cesarean, while the intervention group had 12.7% of surgical deliveries ( *p* = 0.56). Pflugeisen et al. 12 showed a 30.7% rate of cesarean rates in the traditional prenatal care versus 27.4% in telemedicine antenatal care ( *p* = 0.14). 12 The index for preterm birth was evaluated in three of the studies, and the results found were, for the study group versus control group: 3% versus 2.3% ( *p* < 0.71), 7.7 versus 5.8%, and 4 versus 6%, respectively. The incidence of hypertensive diseases of pregnancy were measured in two studies: Pflugeisen et al. 12 and Palmer et al. 14 In both, the percentage of these complications was higher for the intervention with the telemedicine group than in the control group: 8.5 versus 3.4% in the first study, and 9 versus 7% in the second one. None of the studies assessed the numbers of maternal intensive care unit admissions or maternal death. For the fetal outcomes, were assessed neonatal intensive care unit (NICU) admissions, neonatal death, and restricted intrauterine growth (CIUR). The numbers for NICU admissions were identical in the Australian study of 2011 from Palmer et al. 14 : 2% for the group of regular prenatal care versus 2% for the group of telemedicine prenatal care. Pfugleisein et al. find rates of 5.1% in the intervention group versus 7.2% in the control group. 12 The only study that evaluated neonatal death found rates of 1% for each group. 14 The same article was the only one that assessed the rates of CIUR, and found that the rate of babies under percentile 3 of weight for the gestational age was 2% in each group; however, for both data, there was no statistical significance ( *p* = 0.79 for neonatal death and *p* = 0.72 for CIUR).

### Recommendations

**Fig. 3:**
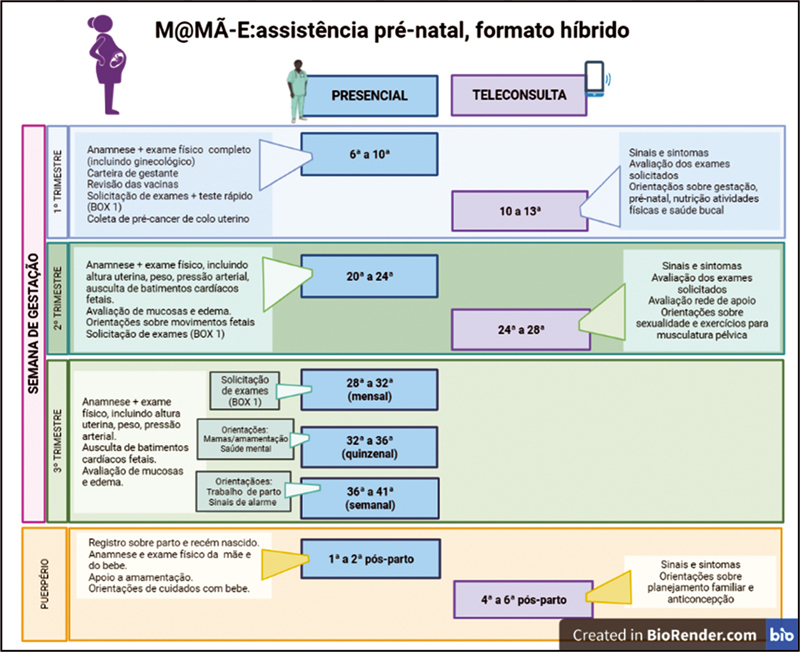
Hybrid low-risk prenatal care guideline schedule for Brazilian pregnant women (Portuguese version).

**Fig. 4:**
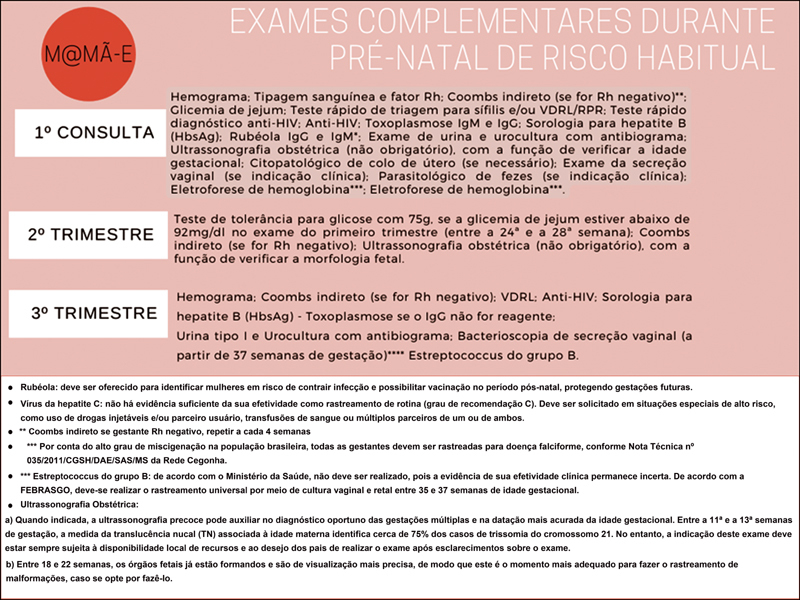
Complementary tests at low-risk prenatal care guideline for Brazilian pregnant women (Portuguese version).

Based on the literature findings, and the most up-to-date recommendations of the Health Ministry and Federação Brasileira das Associações de Ginecologia e Obstetrícia (FEBRASGO), we suggest that a hybrid prenatal care should be included in the guideline for low-risk pregnancy. 5 15 Compliant with Brazilian laws, at least six appointments remain face-to-face and three appointments are offered by telemedicine. The schedule of appointments and complementary exams are shown in Figs. 3 and 4 .

The presented guideline comprises the Ministry of Health recommendations for low-risk prenatal care and reduces exposure to the hospital environment and care costs. A randomized clinical trial, to be developed by this group, will provide real-world data on safety, effectiveness, satisfaction, and costs, one while this review has highlighted those comparative studies that are extremely necessary to evaluate telemedicine antenatal care, especially after the experience that society had in terms of lack of access to essential healthcare, such as prenatal care. In the context of the Sustainable Development Goals (SDG), countries have proposed a new target to accelerate the decline of maternal mortality by 2030. A new model of antenatal care must address inequalities in access, all causes of maternal-fetal mortality and morbidities, and related disabilities and ensure accountability to improve quality of care and equity. 15

## Conclusion

The main strength of this study was a sensitive literature search, without restrictions of data or language to ensure inclusion of all potentially relevant articles. As a limitation, we have only identified a few studies, which provide insufficient data and lead to difficulties in defining a robust recommendation. The present article will help all stakeholders to define a health policy on prenatal care. The new circumstances that have been happening due to the COVID-19 pandemic draw attention to under-recognized health problems and highlight the importance of a new health care model, and preventive interventions. This article is focused on prenatal care of low -risk patients. there is still a need to review prenatal care of those in high-risk groups. This study proposes that services not previously offered to pregnant women are made available as a response to the availability of distant prenatal care, which is safe and less costly.
