# Supplementary material for: Low-Risk Antenatal Care Enhanced by Telemedicine: A Practical Guideline Model
Source: Rev Bras Ginecol Obstet. 2022 Jul 19;44(9):845–53. doi: 10.1055/s-0042-1753505 (PMC9948049; doi:10.1055/s-0042-1753505)
Supplement: Supplementary file 1 — Supplementary Material [file 10-1055-s-0042-1753505-s220033.pdf]

Annex 1 Terminology

Telehealth: Use of electronic and telecommunications technologies to support and promote long-distance clinical health care, patient and professional health-related education, and public health and administration.[2]

Telemedicine: It is a virtual outpatient service, which has gained greater stimulus as a health care model since 2020, due to the COVID-19 pandemic, and it provides a possibility of safe continuous health care, and there will be no need for face-to-face meeting. It is performed through teleconsultations. This methodology favors people with lower purchasing power, people who live long distances from health centers, favors the offer of consultations and decreases the number of people in waiting rooms and receptions.[3] So that health centers can offer quality telemedicine, they need to include in their facilities, quality communication technologies so that they can securely guarantee remote health care.[4]

Teleconsultation: Methodology used to carry out telemedicine. It can be through video calls, phone calls, video conferences and electronic messages.[3,4]

Wearables: Wearable devices (wearables) are revolutionizing biomedicine through mobile and digital health by enabling continuous, longitudinal health monitoring outside of the clinic.[16]

Annex 2

Chart S1 Complete database search

PubMed:
MeSH terms
Entry terms
Termos livres
#1:
Pregnancy [MeSH] OR *Pregnancy* OR *Pregnancies* OR *Gestation* OR *Pregnant Women* [MeSH] OR *Pregnant Women* OR *Pregnant Woman* OR *Woman, Pregnant* OR *Women, Pregnant* OR *Pregnant* OR *Prenatal Care* [MeSH] OR *Prenatal Care* OR *Care, Prenatal* OR *Antenatal Care* OR *Care, Antenatal* OR *Prenatal* OR *Antenatal*
#2:
Telemedicine [MeSH] OR *Telemedicine* OR "*Mobile Health* OR *Health, Mobile* OR *mHealth* OR *Telehealth* OR *eHealth* OR *Remote Consultation* [MeSH] OR *Remote Consultation* OR *Consultation*, *Remote* OR *Teleconsultation* OR *Teleconsultations*
#3: FILTRO ECR
((clinical [Title/Abstract] AND *trial* [Title/Abstract]) OR *clinical trials as topic* [MeSH Terms] OR *clinical trial* [Publication Type] OR *random** [Title/Abstract] OR *random allocation* [MeSHTerms] OR *therapeutic use* [MeSH Subheading])
Cochrane CENTRAL
#1: 22820
MeSH descriptor: [Pregnancy] explode all trees
#2: 337
MeSH descriptor: [Pregnant Women] explode all trees
#3: 1560
MeSH descriptor: [Prenatal Care] explode all trees
#4: 75507
Pregnancy OR *Pregnancies* OR *Gestation* OR *Pregnant Women* OR *Pregnant Woman* OR *Woman, Pregnant* OR *Women, Pregnant* OR *Pregnant* OR *Prenatal Care* OR *Care*, *Prenatal* OR *Antenatal Care* OR *Care*, *Antenatal* OR *Prenatal* OR *Antenatal*
#5: 75749
#1 OR #2 OR #3 OR #4
#6: 2862
MeSH descriptor: [Telemedicine] explode all trees
#7: 401
MeSH descriptor: [Remote Consultation] explode all trees
#8: 9522
Telemedicine OR *Mobile Health* OR *Health, Mobile* OR *mHealth* OR *Telehealth* OR *eHealth* OR *Remote Consultation* OR *Consultation, Remote* OR *Teleconsultation* OR *Teleconsultations*
#9: 9636
#6 OR #7 OR #8
#10: 598
#5 AND #9
Embase:
#1: 1.193.342
pregnancy/exp OR *childbearing* OR *childbearing* OR *gestation* OR *gravidity* OR *intrauterine pregnancy* OR *labor presentation* OR *labor presentation* OR *pregnancy* OR *pregnancy maintenance* OR *pregnancy trimesters* OR pregnantwoman/exp OR *pregnant woman* OR *pregnant women* OR *pregnant* OR *prenatalcare/exp* OR *antenatal care* OR *antenatal care* OR *antenatal control* OR *prenatalcare*
#2: 77.316
telemedicine/exp OR *tele medicine* OR *telemedicine* OR *mobile health*/expOR *telehealth/exp* OR *e-health'* OR *ehealth* OR *tele-health* OR *telehealth* OR *teleconsultation/exp* OR *long distance consultation* OR *remote consultation* OR *tele-consultation* OR *teleconsultation* OR *telephone consultation*
#3: 2.019
#1 AND #2

 © 2022. Federação Brasileira de Ginecologia e Obstetrícia. All rights reserved.
